# The Potential Benefits of HPV E6/E7 mRNA Test in Cervical Cancer Screening in China

**DOI:** 10.3389/fonc.2020.533253

**Published:** 2020-10-02

**Authors:** Shao-Kai Zhang, Zhen Guo, Peng Wang, Le-Ni Kang, Man-Man Jia, Ze-Ni Wu, Qiong Chen, Xiao-Qin Cao, Dong-Mei Zhao, Pei-Pei Guo, Xi-Bin Sun, Jian-Gong Zhang, You-Lin Qiao

**Affiliations:** ^1^Department of Cancer Epidemiology, Affiliated Cancer Hospital of Zhengzhou University, Henan Cancer Hospital, Zhengzhou, China; ^2^Central Laboratory, Affiliated Cancer Hospital of Zhengzhou University, Henan Cancer Hospital, Zhengzhou, China; ^3^Office of Henan Cancer Center, Affiliated Cancer Hospital of Zhengzhou University, Henan Cancer Hospital, Zhengzhou, China; ^4^National Office for Maternal and Child Health Surveillance of China, Department of Pediatrics, West China Second University Hospital, Sichuan University, Chengdu, China; ^5^Key Laboratory of Birth Defects and Related Diseases of Women and Children (Sichuan University), Ministry of Education, Chengdu, China; ^6^Department of Gynecological Oncology, Affiliated Cancer Hospital of Zhengzhou University, Henan Cancer Hospital, Zhengzhou, China; ^7^Department of Cancer Epidemiology, National Cancer Center, Cancer Hospital, Chinese Academy of Medical Sciences and Peking Union Medical College, Beijing, China; ^8^Department of Pathology, Affiliated Cancer Hospital of Zhengzhou University, Henan Cancer Hospital, Zhengzhou, China; ^9^Department of Obstetrics and Gynecology, The Second Affiliated Hospital of Zhengzhou University, Zhengzhou, China

**Keywords:** HPV – human papillomavirus, mRNA, cervical cancer, sensitiviity, specificity

## Abstract

This study aims to evaluate the clinical performance of the HPV E6/E7 mRNA test in cervical cancer screening in China. A hospital-based study was conducted with mRNA, DNA, and liquid-based cytology (LBC) as primary screening tests. Each woman with a positive result received colposcopy with lesion-targeted-biopsy. Histopathological diagnosis was used as the gold standard. The total agreement of HPV DNA and mRNA was 90.7% (95%CI: 87.9, 92.9) with a kappa value of 0.81. The positive rates of HPV DNA, mRNA, and LBC increased with the severity of histopathology diagnosis, from 25.5, 19.1, and 11.4% in normal to 100.0% in SCC, respectively. The sensitivities for mRNA to detect CIN2+ and CIN3+ were 93.8% (95%CI: 89.7–96.4) and 95.7% (95%CI: 91.3–97.9), respectively, which were not different from HPV DNA testing (95.7% [95%CI: 92.0–97.7], 96.3% [95%CI: 92.1–98.3]), but higher than LBC (80.4% [95%CI: 74.5–85.2] and 88.8% [95%CI: 83.0–92.8]). The specificities for mRNA to detect CIN2+ (79.0% [95%CI: 74.2–83.0]) and CIN3+ (70.5% [95%CI: 65.7–74.9]) were higher than HPV DNA testing (71.0% [95%CI: 65.9–75.7], 62.8% [95%CI: 57.8–67.5]), but lower than LBC (84.5% [95%CI: 80.1–88.0] 79.8% [95%CI: 75.4–83.6]). All tests were more effective in women older than 30 years. HPV mRNA test showed excellent agreement with the DNA test, with similar sensitivity and a higher specificity in detecting high-grade cervical lesions. It is promising that mRNA test could be used for the national cervical cancer screening to reduce false positive without losing sensitivity.

## Introduction

It is known that persistent infection with high-risk human papillomavirus (hr-HPV) is a necessary cause for cervical cancer and precancerous lesions (1, 2). With a revealed etiology, cervical cancer is highly preventable (3, 4). Developed countries have begun to use hr-HPV testing in primary cervical cancer screening, either alone or co-testing with cytology (5–8). Evidence shows that HPV-based screening programs provide greater protection against cervical pre-cancer and cancer than other traditional screening methods, such as cytology (9–11). However, since most HPV infections could be cleared spontaneously, HPV-testing identifies numerous infections that will not progress to cervical pre-cancer or cancer, especially in young women (12). Therefore, HPV DNA testing is not recommended to screen women under the age of 30. Moreover, HPV-positive patients referred for subsequent procedures may suffer from unnecessary invasive interventions.

In China, there were 98,900 new cases and 30,500 deaths from cervical cancer in 2015 (13). To curb the increasing trend of this malignancy, the Chinese government has provided a nationwide free cervical cancer screening program for women living in rural areas since 2009, using VIA/VILI, Papanicolaou (Pap) test or HPV test (in pilot sites), based on economic and technological development levels (14). However, due to the nature of these screening methods, the diagnostic accuracy needs to be improved. Moreover, considering China’s large population, novel screening tool with a balance between sensitivity and specificity should be evaluated.

Disease-specific molecular markers of cervical cancer provide a combination of high sensitivity and high specificity to detect cervical pre-cancer. Most of these markers were identified based on the mechanism of HPV-related carcinogenesis. The HPV RNA testing is based on the detection of HR-HPV E6 and E7 mRNA. The oncogenic potential of HPV infection depends on the production of viral E6/E7 oncoproteins. Thus, the detection of E6/E7 mRNA transcripts provides the possibility for a specific test to detect precancerous lesions.

In this study, we evaluated the clinical performance of the HPV E6/E7 mRNA test to detect high-grade cervical intraepithelial neoplasia (CIN) and cancer among Chinese women.

## Materials and Methods

### Participants and Procedures

This hospital-based study was conducted during April to December 2017 in Henan province, China. Women who visited the department of gynecology of The Second Affiliated Hospital of Zhengzhou University (SAHZU) for colposcopy were invited. The inclusion criteria were as follows: (1) women aged between 25 and 64 years-old; (2) no history of cervical cancer or hysterectomy; (3) no clinical symptoms of pregnancy or 8 weeks after the termination of pregnancy; and (4) understand the study procedures, and voluntarily participated. The study was approved by the Institutional Review Board (IRB) of Henan Cancer Hospital (HCH). Written informed consent was obtained from each participant.

The cervical exfoliate cells were obtained from women during the gynecologic examination. The specimen was preserved in 20 ml PreservCyt^®^ transport medium (Hologic Inc., Marlborough, MA, United States) and stored at 4°C. Then, colposcopy examination was performed by a gynecologist. Women with abnormal colposcopy finding underwent lesion-targeted biopsy. If the colposcopy examination was unsatisfactory (the squamocolumnar junction was not completely visible), endocervical curettage (ECC) was performed. Specimens of cervical exfoliated cells were transported to the Cancer Institute and Hospital, Chinese Academy of Medical Sciences (CICAMS). The specimens were divided into two portions: a 1.5 ml cell mixture into a EP tube for the HR-HPV DNA test and HPV E6/E7 mRNA test. The residual preservcyt with exfoliated cells was used for ThinPrep cytologic test (TCT) (Hologic Inc., Marlborough, MA, United States). The Bethesda reporting system was used for cytology by a senior cytologist (15).

### HR-HPV DNA Assay

The 400 μl cell mixture samples from 1.5 ml EP tubes were used for the detection of DNA of 14 hr-HPV types (16, 18, 31, 33, 35, 39, 45, 51, 52, 56, 58, 59, 66, and 68) by Cobas 4800 HPV assay (Roche, Basel, SUI). It reported pooled result of the 14 hrHPV, and the separate result for HPV 16 and 18, simultaneously (16). Cobas 4800 was a PCR-based testing for HPV DNA with nucleic acid hybridization amplification according to the manufacturer’s instructions. Negative and positive quality controls were set in each test. If the ct value was greater than 40, the result was deemed as negative. Otherwise, the result was positive. The positive results included three types: HPV 16, HPV 18, and HPV others (31, 33, 35, 39, 45, 51, 52, 56, 58, 59, 66, and 68).

### HPV E6/E7 mRNA Testing

One ml samples from 1.5 ml EP tubes were used for the detection of the E6/E7 viral mRNA from the 14 hr-HPV types in aggregate APTIMA HPV test (Hologic Inc., Marlborough, MA, United States), according to the manufacturer’s instructions (17). It was a three steps test, including mRNA extraction, amplification, and amplified product detection. If the copy number was greater than or equal to 1.0, the result was deemed as positive. Otherwise, the result was negative.

### Histopathological Diagnosis

Biopsy and ECC tissues were sent to SAHZU for histopathological diagnoses according to the cervical intraepithelial neoplasia (CIN) reporting system. Then histopathologists from HCH reviewed all the slides. Any inconsistent pathological diagnosis was sent to CICAMS for adjudication. The final diagnosis for each woman was based on the worst reading from the panel review. All the detection and diagnosis process were blind.

### Statistical Analysis

The final histopathological diagnosis was used as the gold standard. Women with TCT diagnosis of the low-grade squamous intraepithelial lesion (LSIL) or worse (LSIL +) were deemed as liquid-based cytology (LBC) positive. The HPV DNA results were stratified, according to the HPV types: HPV16/18 (i.e., HPV16 and/or HPV18 positive), HPV-others (i.e., any of the 12 hr-HPV types positive excluding HPV16 and HPV18), and HPV-total (i.e., any of the 14 hr-HPV types positive). The absolute estimates and 95% confidential intervals (95%CI) of positive rates, sensitivity, specificity, positive predictive value (PPV), and negative predictive value (NPV) were calculated. The differences in HPV mRNA expressions in different HPV groups were calculated using the chi-square test or Fisher’s exact test. The McNemar test was used to identify the differences in sensitivity and specificity. The α level was set at 0.05, and *p* < 0.05 (two-sized) was deemed as statistically significant.

## Results

In total, 537 women were included in this analysis. The average age was 43.88 ± 10.97 years. The LBC diagnoses were: 183 (34.7%) normal, 135 (25.1%) atypical squamous cells of undetermined significance (ASC-US), 22 (4.1%) atypical squamous cell cannot exclude HSIL (ASC-H), 7 (1.3%) atypical glandular cell (AGC), 82 (15.3%) LSIL, 75 (14.0%) high-grade squamous intraepithelial lesion (HSIL), 31 (5.8%) squamous cell carcinomas (SCC), and 2 (0.4%) adenocarcinoma *in situ* (AIS). The histopathology diagnoses were 298 (55.5%) normal, 30 (5.6%) cervical intraepithelial neoplasia grade 1 (CIN1), 48 (8.9%) CIN2, 111 (20.7%) CIN3, 43 (8.0%) SCC, and 7 (1.3%) AIS.

Table 1 showed the correlation between mRNA and DNA detection by HPV types. The total agreement of the two tests was 90.7% (95%CI: 87.9, 92.9) with a kappa value of 0.81. The positive rates of mRNA were 91.7%, 84.6%, and 86.4% in HPV16/18, HPV-others, and HPV-total positive women, respectively. All of the mRNA positive rates were higher in HPV-positive women than HPV-negative women in the same HPV type group (all *p* < 0.001).

**TABLE 1:** The correlation of mRNA and DNA detection by HPV types.

**DNA**		**mRNA**				
		**N**	**Positive**	**Negative**	PR (%)*	***P***
HPV16/18	+	168	154	14	91.7	<0.001
	−	369	111	258	30.1	
HPV-others**	+	169	143	26	84.6	<0.001
	−	368	122	246	33.2	
HPV-total***	+	295	255	40	86.4	<0.001
	−	242	10	232	4.1	

*PR: mRNA positive rates by HPV DNA results. **HPV-others: other 12hr-HPV types excluding HPV16 and HPV18. ***HPV-total: 14hr-HPV types in aggregate.

The HPV DNA, mRNA, and LBC positive rates increased with the severity of histopathology diagnosis, ranged from 25.5, 19.1, and 11.4% in normal to 100.0% in SCC, respectively. The positive rates of the three tests in AIS were 85.7, 85.7, and 100.0%, respectively. These three tests had similar positive rates in CIN3, SCC, and AIS. But the positive rates of mRNA were relatively lower in women under CIN2 diagnosis. The rates of HPV16 positive were the highest in CIN3, SCC, and AIS when compared with other HPV DNA groups. HPV18 positive rates were lower than 7% in all squamous cell lesions, but a little higher (28.6%) in AIS (Table 2).

**TABLE 2:** HPV positive rates by the severity of histopathology diagnosis.

**Histopathology diagnosis**	**DNA (%)**				**mRNA (%)**	**LBC (%)**
	**HPV 16**	**HPV 18**	**HPV-others**	HPV-total**		
Normal (*n* = 298)	16 (5.4)	8 (2.7)	59 (19.8)	76 (25.5)	57 (19.1)	34 (11.4)
CIN1 (*n* = 30)	7 (23.3)	1 (3.3)	11 (36.7)	19 (63.3)	12 (40.0)	17 (56.7)
CIN2 (*n* = 48)	15 (31.3)	3 (6.3)	34 (70.8)	45 (93.8)	42 (87.5)	25 (52.1)
CIN3 (*n* = 111)	79 (71.2)	3 (2.7)	50 (45.0)	106 (95.5)	105 (94.6)	94 (84.7)
SCC (*n* = 43)	34 (79.1)	1 (2.3)	14 (32.6)	43 (100.0)	43 (100.0)	42 (97.7)
AIS (*n* = 7)	4 (57.1)	2 (28.6)	1 (14.3)	6 (85.7)	6 (85.7)	7 (100.0)
Total	155 (28.9)	18 (3.4)	169 (31.5)	295 (54.9)	265 (49.3)	219 (40.8)

*HPV-other: other 12 hr-HPV types excluding HPV16 and HPV18. **HPV-total: 14hr-HPV types in aggregate.

Table 3 showed the clinical performance of the three tests in detecting CIN2+ and CIN3+ lesions. The sensitivities for mRNA to detect CIN2+ and CIN3+ were 93.8% (95%CI: 89.7–96.4) and 95.7% (95%CI: 91.3–97.9), respectively, which were not different from those detected by DNA (95.7% [95%CI: 92.0–97.7] for CIN2+, 96.3% [95%CI: 92.1–98.3] for CIN3+), but higher than those detected by LBC (80.4% [95%CI: 74.5–85.2] for CIN2+ and 88.8% [95%CI: 83.0–92.8] for CIN3+). The specificities for mRNA to detect CIN2+ (79.0% [95%CI: 74.2–83.0]) and CIN3+ (70.5% [95%CI: 65.7–74.9]) were significantly higher than those detected by DNA (71.0% [95%CI: 65.9–75.7] for CIN2+, 62.8% [95%CI: 57.8–67.5] for CIN3+) (*p* < 0.05), but lower than those detected by LBC (84.5% [95%CI: 80.1–88.0] for CIN2+, 79.8% [95%CI: 75.4–83.6] for CIN3+). The PPVs for mRNA to detect CIN2+ and CIN3+ were 74.0% (95%CI: 68.4–78.9) and 58.1% (95%CI: 52.1–63.9), and the NPVs were 95.2% (95%CI: 92.0–97.2) and 97.4% (95%CI: 94.8–98.8), respectively.

**TABLE 3:** The clinical performance for tests to detect CIN2+ and CIN3+ lesions (%).

**Endpoints**	**Tests**	**Sensitivity (95%CI)**	**Specificity (95%CI)**	**PPV (95%CI)**	**NPV (95%CI)**
CIN2+	mRNA	93.8 (89.7, 96.3)	79.0 (74.2, 83.0)	74.0 (68.4, 78.9)	95.2 (92.0, 97.2)
	DNA	95.7 (92.0, 97.7)	71.0 (65.9, 75.7) ^*a*^	67.8 (62.3, 72.9)	96.3 (93.1, 98.0)
	LBC	80.4 (74.5, 85.2)	84.5 (80.1, 88.0)	76.7 (70.7, 81.8)	87.1 (83.0, 90.4)
	**X^2^**	33.84	17.67	5.55	22.12
	*P*	<0.001	<0.001	0.06	<0.001
CIN3+	mRNA	95.7 (91.3, 97.9)	70.5 (65.7, 74.9)	58.1 (52.1, 63.9)	97.4 (94.8, 98.8)
	DNA	96.3 (92.1, 98.3)	62.8 (57.8, 67.5) ^*a*^	52.5 (46.9, 58.2) ^*a*^	97.5 (94.7, 98.9)
	LBC	88.8 (83.0, 92.8)	79.8 (75.4, 83.6)	65.3 (58.8, 71.3)	94.3 (91.2, 96.4)
	**X^2^**	9.17	27.45	8.78	5.33
	*P*	0.01	<0.001	0.01	0.07

*PPV, positive predictive value; NPV, negative predictive value; a means compared with RNA, *p* < 0.05.*

Tables 4, 5 showed the positive rates and clinical performance of the three tests in different age groups. In summary, all the tests showed better performance for women older than 30 years than younger women. HPV DNA test had the highest sensitivity, and LBC had the highest specificity for detecting CIN2+ and CIN3+ in women younger than 30 years.

**TABLE 4:** The positive rates and clinical performance of tests to detect CIN2+ in different age groups.

**Tests**	**PR (95%CI)**	**Sensitivity (95%CI)**	**Specificity (95%CI)**	**PPV (95%CI)**	**NPV (95%CI)**
≤30					
mRNA	43.1 (32.3, 54.6)	81.8 (61.5, 92.7)	74.0 (60.5, 84.1)	58.1 (40.8, 73.6)	90.2 (77.5, 96.1)
DNA	54.2 (42.7, 65.2)	90.9 (72.2, 97.5)	62.0 (48.2, 74.1)	51.3 (36.2, 66.1)	93.9 (80.4, 98.3)
LBC	36.1 (26.0, 47.7)	72.7 (51.9, 86.9)	80.0 (67.0, 88.8)	61.5 (42.5, 77.6)	87.0 (74.3, 93.9)
X^2^		2.38	3.39	0.52	1.05
*P*		0.35	0.18	0.77	0.64
31–45					
mRNA	47.8 (41.4, 54.3)	94.4 (87.5, 97.6)	82.0 (74.8, 87.5)	77.1 (68.3, 84.0)	95.8 (90.5, 98.2)
DNA	53.9 (47.5, 60.3)	95.5 (89.0, 98.2)	72.7 (64.7, 79.4)	69.1 (60.5, 76.6)	96.2 (90.6, 98.5)
LBC	40.4 (34.2, 46.8)	83.2 (74.0, 89.5)	87.1 (80.5, 91.7)	80.4 (71.2, 87.3)	89.0 (82.6, 93.2)
X^2^		11.73	10.75	4.65	7.64
*P*		0.003	0.005	0.098	0.02
>45					
mRNA	52.7 (46.4, 59.0)	95.9 (90.0, 98.4)	77.7 (70.1, 83.8)	75.2 (67.0, 81.9)	96.4 (91.2, 98.6)
DNA	56.1 (49.8, 62.3)	96.9 (91.4, 99.0)	72.7 (64.7, 79.4)	71.4 (63.2, 78.4)	97.1 (91.9, 99.0)
LBC	42.6 (36.5, 49.0)	79.6 (70.6, 86.4)	83.5 (76.4, 88.7)	77.2 (68.1, 84.3)	85.3 (78.4, 90.3)
X^2^		22.27	4.71	1.08	15.53
*P*		<0.005	0.10	0.58	<0.001

**TABLE 5:** The positive rates and clinical performance of tests to detect CIN3+ in different age groups.

**Tests**	**PR (95%CI)**	**Sensitivity (95%CI)**	**Specificity (95%CI)**	**PPV (95%CI)**	**NPV (95%CI)**
≤30					
mRNA	43.1 (32.3, 54.6)	86.7 (62.1, 96.3)	68.4 (55.5, 79.0)	41.9 (26.4, 59.2)	95.1 (83.9, 98.7)
DNA	54.2 (42.7, 65.2)	93.3 (70.2, 98.8)	56.1 (43.3, 68.2)	35.9 (22.7, 51.6)	97.0 (84.7, 99.5)
LBC	36.1 (26.0, 47.7)	80.0 (54.8, 93.0)	75.4 (62.9, 84.8)	46.2 (28.8, 64.5)	93.5 (82.5, 97.8)
X^2^		1.18	4.12	0.54	0.57
*P*		0.86	0.13	0.76	0.88
31–45					
mRNA	47.8 (41.4, 54.3)	96.1 (89.2, 98.7)	76.8 (69.5, 82.8)	67.9 (58.6, 75.9)	97.5 (92.9, 99.1)
DNA	53.9 (47.5, 60.3)	96.1 (89.2, 98.7)	67.6 (59.7, 74.5)	60.2 (51.3, 68.4)	97.1 (91.9, 99.0)
LBC	40.4 (34.2, 46.8)	87.0 (77.7, 92.8)	83.4 (76.7, 88.5)	72.8 (63.0, 80.9)	92.7 (87.0, 96.0)
X^2^		6.58	12.43	4.87	4.23
*P*		0.04	0.002	0.09	0.12
>45					
mRNA	52.7 (46.4, 59.0)	97.1 (90.0, 99.2)	65.5 (58.0, 72.3)	53.6 (44.9, 62.1)	98.2 (93.7, 99.5)
DNA	56.1 (49.8, 62.3)	97.1 (90.0, 99.2)	60.7 (53.2, 67.8)	50.4 (42.0, 58.7)	98.1 (93.3, 99.5)
LBC	42.6 (36.5, 49.0)	92.8 (84.1, 96.9)	78.0 (71.1, 83.6)	63.4 (53.6, 72.1)	96.3 (91.7, 98.4)
X^2^		1.84	12.28	4.10	1.12
*P*		0.52	0.002	0.13	0.57

*PR, positive rate; PPV, positive predictive value; NPV, negative predictive value.*

## Discussion

The presented results showed that the mRNA test and DNA teat reached a high agreement as 90.7%. The HPV DNA, mRNA, and LBC positive rates increased with the severity of histopathology diagnosis, from 25.5, 19.1, and 11.4% in normal to 100.0% in SCC, respectively. The sensitivities for mRNA to detect CIN2 and CIN3+ were 93.8% and 95.7%, which were similar to those detected by DNA (95.7% for CIN2+ and 96.3% for CIN3+). But the specificities for mRNA (79.0% for CIN2+ and 70.5% for CIN3+) were significantly higher than those detected by DNA (71.0% for CIN2+ and 62.8% for CIN3+).

In this study, the HPV E6/E7 mRNA test and HPV DNA test showed high agreement. Of those HPV 16/18 DNA positive women, 91.7% were also positive on mRNA, which is similar to other studies, that reported an overall agreement of over 90% between APTIMA HPV test and HPV DNA tests (17, 18), and consistently higher positive rates for HPV DNA test in different populations (19, 20). We noticed higher positive rates in older women than younger women, which was slightly different from other studies (21–23). This variation may be attributed to the study design (i.e., hospital-based study), which limits the extrapolation of the findings into the general population. Interestingly, the discordant rate was higher in women aged 30 years or younger (25.6% vs. 8.6%). Studies have demonstrated a higher rate of spontaneous clearance for HPV infection in younger women, which indicated a lower possibility of HPV integration (24).

As we know, DNA-based HPV tests detect the presence or absence of HPV DNA. However, most HPV infections are transient, cleared spontaneously within 1 year, which would not progress to cervical pre-cancer or cancer (25). E6/E7 mRNA expression only occurs in actively infected cells and increase during CIN development and progression (26). Therefore, the HPV mRNA test is supposed to be more specific in detecting high-grade cervical lesions. Our data confirmed this hypothesis. We found that the mRNA test was as sensitive as the DNA test, but more specific in detecting cervical pre-cancer and cancer. Other researchers drew similar conclusions in either primary screening (20) or triage of women with minor abnormal cytology (27). In our study, 50 women had discordant results between HPV DNA and mRNA test. Among them, 34 DNA + /mRNA- versus 8 mRNA + /DNA- were diagnosed as normal or CIN1; 6 versus 2 were diagnosed as CIN2 or CIN3; no test difference was observed in cancers. Therefore, if we replace the HPV DNA test with the mRNA test, 34 women would not be referred for unnecessary colposcopy, but 4 CIN2 and 2 CIN3 would be missed. In spite of 6 cases missing, there was no sensitivity lost, but specificity increased. Literature showed 40–60% of CIN2 cases would regressed within 2 years (28, 29), and not all CIN3 cases were true pre-cancerous lesions (30). Cook et al. also found a low CIN2+ rate among mRNA-/DNA + women (17). Therefore, the risk of invasive cancer for the 6 women was low in a short time interval.

We also evaluated the test performance in older and younger women. The age-specific analysis found that the three tests functioned better in women older than 30 years. Based on the fact that younger women with CIN lesions had a higher possibility of regression, we suggest a conservative management of younger women. Studies have found that HPV infection was common in young women, however, a large portion of them were transient infections, and may be cleared spontaneously (31, 32). The short duration of most HPV infections in these women suggests that the associated cervical dysplasia should be managed conservatively (12). Therefore, the HPV DNA test may not be a suitable screening method for women under 30 years of age, due to the high false-positive rate. The screening performance of cytology and E6/E7 mRNA comprehensively in young women should be evaluated. In our study, data showed that cytology had the highest specificity in women under 30 years, but the sensitivity is lower than the HPV DNA test. However, for E6/E7 mRNA, it had a moderate performance in women younger than 30 years, which had higher sensitivity than cytology accompanied by higher specificity than HPV DNA, suggesting that E6/E7 mRNA testing may be a promising option for the screening of women under 30.

Although the China Food and Drug Administration approved three prophylactic HPV vaccines (i.e., Cervarix, Gardasil, and Cecolin), cervical cancer prevention still relies on screening. In 2009, the Chinese government launched a nationwide free cervical cancer screening for rural women. In 2015, the HPV DNA test was used as a primary screening tool in pilot sites for the first time. However, due to its large population, HPV DNA-based screening would result in unavoidable huge false positives, leading to a waste of health resources and unnecessary anxiety. It is promising that mRNA could be used in the national cervical cancer screening program to reduce the false positive without losing any sensitivity.

Several limitations should be addressed in this study. First, this is a cross-sectional study, and we could not evaluate the risk of lesion progression associated with HPV E6/E7 mRNA. However, we stratified our data by histological grade, which provides information on the correlation. Second, it is important to note that the results cannot be fully generalized to the general population because the study participants were recruited from the outpatient in hospital.

In conclusion, the APTIMA mRNA test had good agreement with Cobas 4800 HPV DNA test with similar sensitivity and higher specificity to detect high-grade cervical lesions. It is promising that mRNA could be used in China’s national cervical cancer screening program to reduce the false positive without losing any sensitivity. Further studies are needed to evaluate the clinical performance of mRNA test in younger women in China.

## Data Availability Statement

All datasets generated for this study are included in the article/supplementary material.

## Ethics Statement

The studies involving human participants were reviewed and approved by the role of co-expression of HPV E6/E7 mRNA and p16/Ki-67 protein in predicting the risk of cervical cancer. By Life Science Ethics Review Committee of Zhengzhou University. The patients/participants provided their written informed consent to participate in this study.

## Author Contributions

S-KZ contributed to the design and wrote the manuscript. ZG contributed to the manuscript’s writing and revision. PW, M-MJ, and P-PG contributed to the investigation and HPV DNA test. L-NK and Z-NW contributed the HPV mRNA test. D-MZ contributed to cytology and histology examination. QC and X-QC performed the statistical analysis. X-BS helped to conceive the study and assisted with the statistical analyses. Y-LQ and J-GZ helped to conceive the study and assisted with the manuscript’s writing. J-GZ assisted with the implementation of the study. All authors read and approved the final manuscript.

## Conflict of Interest

The authors declare that the research was conducted in the absence of any commercial or financial relationships that could be construed as a potential conflict of interest.

## Abbreviations

hr-HPV: high-risk human papillomavirus
VIA/VILI: visual inspection with acetic acid/visual inspection with Lugol’s Iodine solution
Pap: papanicolaou
SAHZU: Second Affiliated Hospital of Zhengzhou University
IRB: Institutional Review Board
HCH: Henan Cancer Hospital
CICAMS: Cancer Institute and Hospital Chinese Academy of Medical Sciences
95%CI: 95% confidential intervals
ECC: endocervical curettage
TCT: ThinPrep cytologic test
CIN: cervical intraepithelial neoplasia
LSIL: low-grade squamous intraepithelial lesion
HSIL: high-grade squamous intraepithelial lesion
LBC: liquid-based cytology
PPV: positive predictive value
NPV: negative predictive value
ASC-US: atypical squamous cells of undetermined significance
ASC-H: atypical squamous cell cannot exclude HSIL
AGC: atypical glandular cell
SCC: squamous cell carcinomas
AIS: adenocarcinoma *in situ*.
